# Good Deeds Could Come From Frustrated Individuals

**DOI:** 10.3389/fpsyg.2021.631711

**Published:** 2021-05-04

**Authors:** Yibo Peng, Jinghua Tang, Hanzhou Li

**Affiliations:** School of Medicine and Holistic Integrative Medicine, Nanjing University of Chinese Medicine, Nanjing, China

**Keywords:** frustration, altruistic behavior, mood, donation behavior, emotion induction

## Abstract

Frustration is often seen as negative, but as to whether it may have a positive impact on the individual is still undecided. This research was conducted to explore the influence of frustration on altruistic tendency and altruistic level in college students (17–21 years old). By presenting a highly difficult task combined with negative feedback, we effectively induced frustration in Experiment 1 (*n* = 70). By assessing the donation behavior of participants (*n* = 54) in a real-life scenario following the experimental manipulation of frustration, we examined the relationship between frustration and altruism in Experiment 2. Results showed that frustrating situations could, on some level, improve altruistic behavior [*t*_(8.834)_ = 3.013, *p* = 0.015]. More specifically, among participants who donated, the amount donated was higher in the frustration group compared to the control (fulfillment) group; the proportion of people who donated did not differ by group.

## Introduction

An individual encounters a frustrating situation when they are confronted by internal or external obstacles preventing them from completing a specific purposeful activity (Lin, 2017). This can lead to the emotion of frustration, which may affect an individual’s behavioral decisions. It has been demonstrated empirically that negative outcomes can occur after an individual encounters a frustrating situation. The Frustration-Aggression theory by Dollard and colleagues proposes that aggression is usually the result of frustration, and frustration may lead to some form of aggression (Dollard et al., 1939; Berkowitz, 1989). Many researchers have tried to test as well as reformulate this theory. Berkowitz (1989) proposed that frustration does not always lead to aggression. There is no denying that frustrating events have a negative impact on the individual, but the question is, does frustration in addition to negative consequences (i.e., aggression), in the short term, also have a positive effect on individuals?

Frustration has a lot in common with trauma. For example, both are associated with negative effects (i.e., arouse anger or sadness) in individuals in the short term. Nonetheless, the response to trauma, although typically a negative state, can have positive influences on behavior in the long term. Frustration, as a negative emotional state, could possibly have the same effect. With the rise of positive psychology in recent years, more researchers have turned their attention to the positive impact of trauma on individuals. Tedeschi and Calhoun (1996) proposed the concept of post-traumatic growth (PTG) and defined it as a positive change in psychological aspects experienced after overcoming a traumatic event or situation. After reviewing and summarizing a large body of previous evidence, Staub and Vollhardt (2008) put forward a conceptual model of “altruism born of suffering.” This model considers the reasons for the increase in the altruistic behavior tendency of individuals after experiencing negative events from the perspective of internal and external factors (Staub and Vollhardt, 2008; Tu and Guo, 2010). The model proposes that an individual’s negative experience will increase their level of empathy and produce altruistic behavior. Clark (1991) believed that altruism can create or enhance the meaning of life. Through altruism, individuals can improve their sense of self-efficacy, enhance their cognitive ability, and actively interpret suffering, and therefore, better cope with suffering themselves (Clark, 1991). From this perspective, altruistic behavior is not only a result of PTG but also a factor that promotes the growth of individuals after trauma.

O’Leary and Ickovics (1995) believed that despite the same or similar setbacks, different changes in individuals’ physical and/or psychological states may result in at least four different outcomes. The first is that the individual’s physical and/or psychological state(s) decline continuously and eventually dysfunction. The second is that certain physical or mental functions of the individual are impaired, and the third is that the individual’s functions are impaired but then gradually return to the previous level. The fourth is that the individual’s functions not only return to the previous level but even exceed the original level in some respects (O’Leary and Ickovics, 1995; Carver, 1998).

Therefore, the impact of frustration on individuals can be complicated. In an individual’s daily life, frustration tends to not be serious enough to cause permanent dysfunction or significant growth. Two outcomes, based on the theory of O’Leary and Ickovics (1995), are most likely to occur: First, after experiencing frustration, individuals would develop cognitive dysfunction, and therefore be only negatively affected. Second, after experiencing frustration, while individuals would experience negative emotions, other aspects would prompt positive changes to some extent.

The second situation described above may be similar to the concept of PTG, as both express positive changes produced by individuals after negative situations. One of the major manifestations of PTG is the enhancement of an individual’s empathy ability, leading them to be more likely to engage in altruistic behavior when facing other people’s misfortune (Tedeschi and Calhoun, 1996). Therefore, if a frustrating situation increases the participants’ altruistic behavior tendency or level, in a similar way to PTG, then the reason for this change in the individual could be attributed to the fact that the frustrating situation had improved the individual’s empathy ability.

Thus, what is the impact of frustration on altruistic behavior? There are many factors related to altruism. For example, emotional state may influence tendency toward altruistic behavior. In the experiment by Wang (2017), participants in a positive emotional state were more likely to perform altruistic behaviors, and those in a negative emotional state were less likely to perform altruistic behaviors. Nonetheless, positive change in the form of PTG is possible after an individual has experienced trauma (Tedeschi and Calhoun, 1996), and an increase in altruistic behavior tendency is a typical change of PTG (Staub and Vollhardt, 2008). Moreover, many studies have shown that empathy is an important factor that can lead to altruistic behavior (Batson et al., 1981). According to the Negative State Relief Hypothesis, when witnessing the suffering of others, an individual who has experienced suffering will feel more sympathetic pain and therefore will engage in altruistic behavior to relieve or alleviate the suffering of others (Cialdini et al., 1987). Although the empathy level and altruistic tendency increase with the perceived similarity to the victim’s experience (Dovidio et al., 1997; Lv and Huangfu, 2018), frustrated individuals are also likely to engage in a more general form of altruism because other kinds of suffering can also be interpreted as a “common destiny” similar to one’s own (Lv and Huangfu, 2018). Empathy often reaches beyond its original evolutionary context, which means that an individual may empathize with the sufferer and act altruistically while ignoring the sufferer’s characteristics and the details of one’s own suffering (De Waal, 2008; Lv and Huangfu, 2018). There is thus sufficient evidence that empathy not only can trigger an individual’s altruistic behavior toward sufferers with similar experiences, but also a more general altruism.

A change in empathy levels of individuals in frustrating situations may be paralleled with the change in empathy levels of individuals who have experienced trauma because negative experiences could push individuals to more easily empathize with others who experience negative events. However, the severity (duration, magnitude of effects) of trauma and frustration are different; and consequently, the degree and duration of individual “growth” will also be different. If frustration can induce a positive change in an individual and increase the level of empathy, is the change strong enough to increase the individual’s tendency to engage in altruistic behavior? Thus, Experiment 2 explored the issue of whether frustration can bring a positive change to individuals, increasing their tendency to do good deeds.

A large number of studies have found that the most effective way to induce emotions is to trigger them using corresponding emotionally charged materials (Li, 2016). Therefore, one effective way to induce frustration in individuals is to create an effective frustrating situation. Researchers often induce frustration by artificially creating negative feedback in experiments (Lin, 2017) or hindering participants from achieving success (Henna et al., 2008). However, this kind of experimental operation is sometimes too deliberate and can be discovered by the participants. For example, participants may be confused when they actually answer a question correctly but receive feedback that they have provided a “wrong answer,” or they may question why they are always hindered at the end of the experimental task. Therefore, in this study, time-limited and difficult tasks were arranged, and real feedback was given to the answer for each question. The preset negative feedback was given in the three summaries and the final results of the experiment to reduce the face validity of the frustration-inducing task. Experiment 1 showed that presenting a highly difficult task combined with negative feedback induced frustration effectively.

As an increase in altruistic behavioral tendencies is one of the important manifestations of PTG, altruistic behavioral tendencies were the dependent variable in Experiment 2 in this research to allow exploration of the relationship between frustration and altruistic behaviors. In other words, this research attempted to establish whether frustration can promote the occurrence of a short-term positive change similar to PTG in the individuals experiencing it. Hence, we predicted that an experimentally induced frustrating situation would improve the tendency and level of altruistic behavior in participants.

The current study is also related to dual-process theory, which refers to a set of frameworks sharing the core idea that people’s choices result from the interplay between two cognitive systems, one (System 1) that is fast and intuitive and one (System 2) that is slow and deliberative (Capraro, 2019). In this case, when an individual chooses to use the second strategy, more cognitive resources are needed (Evans and Stanovich, 2013). In the current experiment, the highly difficult task was used to induce participants’ frustration, but the difficult task may inhibit the use of System 2 because the limited working memory, which is highly needed by System 2 (Evans and Stanovich, 2013; Capraro, 2019), was partly taken up by the difficult task (Capraro, 2019). In addition, the frustration-inducing task may also function as an experimental manipulation called “ego depletion,” which aims to deplete cognitive resources (Fromell et al., 2020). Thus, according to the meta-analysis conducted by Fromell et al. (2020), in the current study, participants in the frustration group were under a greater “cognitive load” and may have been less able to use their deliberation system (System 2) and more likely to use their intuition system (System 1). This resulted in conducting a higher level of altruistic behavior, because they had few cognitive resources left to consider their self-interest. This view can also support the argument that good deeds conducted by a frustrated individual cannot be as significant as PTG, because, relatively speaking, behavior promoted by a frustrating situation is quick and intuitive (System 1). Nonetheless, it remains controversial whether altruism is an intuitive or deliberative choice (Fromell et al., 2020). Although both the predictions from the General Social Heuristics Hypothesis (Capraro, 2019) and the meta-analysis results (Fromell et al., 2020) indicate that dual-process strategies and altruism are unrelated, it is still interesting to explore which factors led 57% of the 60 studies to find self-interest as an intuitive response while the rest came to the opposite conclusion (Fromell et al., 2020). The behavior the individual chooses when they are experiencing both frustration and being motivated to choose System 1 as strategy toward altruism is discussed in the current study.

Many studies have been conducted on the measurement of altruistic behavior. There are three common methods: questionnaires, observational, and behavioral paradigms (Dong, 2019; Filkowski et al., 2016). Rushton et al. (1981) developed the self-report altruism scale, which consists of a total of 20 items about altruistic behaviors that often occur in people’s daily lives. This scale is prevalently used to measure altruistic personality. Nevertheless, self-report measures tend to have the risk of social desirability effects. Altruistic behavior, rather than altruistic personality, was the focus of the current study. Thus, questionnaires were not suitable. As for the behavioral paradigms, actual behaviors within a social situation are measured by the participants’ reactions to a simulated situation in the laboratory (Filkowski et al., 2016). For example, the dictator game, in which the participant performs as a dictator and must decide how much of the money received from the experimenter to donate to the recipient, is an effective method to measure altruism (Capraro, 2019). However, in the circumstance where the cost of helping is smaller than the benefit, social desirability effects may be a confounding factor (Capraro, 2019). Using the observation method, the tendency of individuals to do good deeds is observed and recorded under laboratory or natural conditions (Dong, 2019). Given that we intended to measure state (immediate) rather than trait (personality-based) altruism, which is relatively stable, we used observation.

After choosing observation to measure altruistic behavior, a specific altruistic behavior of interest was chosen that could appropriately reflect the individual’s altruistic behavior tendency and its level. Baum (2001) suggests that charitable donation, which refers to the behavior of giving others money, goods, or services voluntarily, is a typical form of altruistic behavior. The theory of planned behavior holds that “behavior intention is the most direct factor affecting behavior, and behavior intention is in turn influenced by attitude, subjective norm, and perceived behavior control” (Duan and Jiang, 2008). This theory originated from Fishbein’s theory of multi-attribute attitude (Fishbein, 1963) and has been improved and developed into a set of theoretical models with significant explanatory and predictive power in terms of behavior (Duan and Jiang, 2008). According to this theory, the decision about donating in individuals is mainly affected by altruistic behavior tendency (Xie, 2013); that is to say, the higher the individual’s altruistic behavior tendency is, the greater the likelihood of donation behavior. Therefore, whether to donate reflects the individual’s tendency toward altruistic behavior; and the donation amount *per se* can reflect the level of altruistic behavior. In short, donation behavior can accurately reflect an individual’s altruistic behavior tendency and level, and in this research, the form of fund-raising participant fees was used.

Nonetheless, individuals tend to behave or claim to be more in line with social expectations when conducting donation experiments (Bekkers and Wiepking, 2011). To avoid the influence of the social desirability effect in the observation method, the donation experiment was based on a real scenario. Based on the literature discussed, for Experiment 2 it was predicted that the participants who experienced larger amounts of frustration would be more likely to display donation behavior.

In summary, Experiment 1 tested the effectiveness of a frustration-inducing procedure. It was predicted:

Hypothesis 1a: Accuracy would be lower in the frustration task than in the fulfillment task.
Hypothesis 2a: Participants in the frustration group would have higher scores in the F, PS and AH dimensions of CMACL, and a lower score in the HE dimension of CMACL.
Hypothesis 3a: Participants in the frustration group would have higher self-reported frustration and lower self-reported fulfillment.

Experiment 2 tested the effects of frustration on altruistic tendency and level. It was predicted:

Hypothesis 1b: Participants in the frustration group are more likely to display donation behavior (altruistic tendency) than those in the fulfillment group.
Hypothesis 2b: The number of participants who displayed donation behavior (altruistic level) in the frustration group would be higher than the number in the fulfillment group.

## Experiment 1: A Test of the Effectiveness of the Frustration-Inducing Procedure

### Method

The ethics committee at the sponsoring university approved all procedures used in the current study. Informed consent was obtained from participants after thoroughly explaining the nature and consequence of this study. The participants have consented to the submission of the case report to the journal.

#### Participants

A total of 88 undergraduate students (17–21 years old, *M* = 18.60 years, *SD* = 0.79 years) from Nanjing University of Chinese Medicine (63 in the frustration group and 25 in the fulfillment group) were enrolled. Participants were randomly assigned to groups. Data from participants who repeated the same choice more than five times or quitted the experiment halfway were deleted. Seventy participants were valid (48 vs. 22), which resulted in 19 males in the frustration group and 8 males in the fulfillment group.

#### Procedure

A between-subjects design was adopted. The independent variable was the combination of artificial feedback and task difficulty. There were two levels: a high difficulty task with negative feedback (frustration group) and a low difficulty task with positive feedback (fulfillment group). The task used was the same between groups except for the task difficulty and feedback. The dependent variables included the self-rated scores of the four dimensions of the Chinese Mood Adjective Check List (CMACL) and the self-rated scores for frustration and fulfillment.

Experiment 1 was programmed and conducted using E-prime 2.0.8, and statistical analysis of data was performed using SPSS 22.0 software. Experiment 1 included the two parts described below.

#### Frustration-Inducing Experimental Task

The frustration-inducing experimental paradigm in this experiment used questions from the 2018 and 2019 prefecture-level and vice-province Administrative Aptitude Test of the Chinese National Civil Service Examination (which is used to select civil servants in China) and was adapted from that used by Lin (2017). Questions were divided into three types according to their accuracy (National Public Servant, 2019): difficult (accuracy rate lower than 30%), medium (accuracy rate between 30% and 70%), and simple (accuracy rate higher than 70%). In the frustration-inducing task, three types of skills were tested: mathematical ability (e.g., solving real problems through equations), language comprehension ability (e.g., competing sentences with the appropriate words), and logical ability (e.g., analogical reasoning problems).

Before the experiment began, text was displayed on the screen to inform participants that the “Comprehensive Cognitive Ability Test” would be completed, that it would include three parts (mathematical ability, language comprehension ability, and logic ability), and that each part would have 10 questions.

Each question was completed within 90 s followed by reaction time and feedback information displayed on the screen for 500 ms, both of which were genuine. If the participants made no response during the 90 s, the feedback “Time out” in red font was displayed on the screen. Blue font indicated that the participant answered correctly, and red font indicated that the answer was incorrect. The setting of the font color served the purpose of allowing participants to build a connection between the color and correctness.

At the beginning of each part, participants were asked to evaluate their corresponding abilities (i.e., the percentage of their peers they believed they could exceed). At the end of each test, preset feedback (depending on which experimental group the participant belonged to) was presented on the screen (i.e., the percentage of their peers they exceeded). After completing all the tasks, the overall performance for the “Comprehensive Cognitive Ability Test” was conveyed on screen. These results were also preset depending on which group participants were allocated to, including the positive and negative feedback.

For the frustration group, the difficulty ratio of questions in each part was 3:3:4 (simple: medium: difficult), and participants received preset negative feedback after each part, namely, “Your mathematical ability/language comprehension ability/logic ability exceeds 14.7%/12.4%/13.6% of peers,” presented in red font. After completing all the tasks, the following feedback was displayed in red font on the screen: “Your Comprehensive Cognitive Ability: Poor.”

For the fulfillment group, the difficulty ratio of questions in each part was 4:4:2 (simple: medium: difficult), and participants received preset positive feedback after each part, namely, “Your mathematical ability/language comprehension ability/logic ability exceeds 83.7%/92.4%/87.1% of peers,” presented in blue font. After completing all the tasks, the following feedback was displayed in blue font on the screen: “Your Comprehensive Cognitive Ability: Excellent.”

#### Effectiveness of Frustration-Inducing Experimental Task

After the participants completed the experimental task, they evaluated their emotional state, frustration, and fulfillment. The mood adjective check list is a psychological measurement of mood commonly used in clinics (Zhong and Qian, 2005). Zhong and Qian (2005) compiled the CMACL based on the emotional structure identified in Chinese people and the specificity of Chinese. The scale contains four dimensions: Fidgeting (F), Happy and Excited (HE), Pained and Sad (PS), Angry and Hating (AH), and assessed in a total of 30 items. It asks individuals to rate each adjective from 1 to 5 (1, completely inconsistent; 2, partially inconsistent; 3, uncertain; 4, partially consistent; 5, completely consistent) according to how they felt at that time. The consistency reliability and split-half reliability of each dimension of the scale are above 0.8, and the qualified reliability and validity mean it can be used as an assessment tool for frustration induction through the assessment of related emotions (Zhong and Qian, 2005).

Participants completed CMACL after the “Comprehensive Cognitive Ability Test” in Experiment 1. The items were presented in random order to avoid sequential effects. After completing the scale, the participants were asked to self-evaluate the frustration and fulfillment they felt at the end of the experiment. The instruction was: “After completing the *Comprehensive Cognitive Ability Test*, how much frustration/fulfillment did you experience?” The presentation order of frustration/fulfillment was randomly determined, and participants responded on a five-point Likert scale to provide a subjective assessment of frustration and fulfillment.

#### Debrief

Immediately after the experiment, the researchers debriefed the participants on the true purpose of the experiment to comply with the relevant ethics (according to 1964 Declaration of Helsinki and its later amendments or comparable ethical standards). For the participants who quit the experiment voluntarily halfway, we immediately conducted the debriefing.

We debriefed the participants on the following:

(1) The performance feedback for every participant was preset, and the results do not reflect any ability of the participants.
(2) The so-called “Comprehensive Cognitive Ability Test” in this experiment was fabricated by the researchers to induce specific emotional states.
(3) The real purpose of the experiment was to discover whether the paradigm can induce specific emotional states in participants.

Considering that negative emotion can be induced during such experiments, the researchers provided the participants with some emotional comfort (i.e., verbal comfort (encourage their abilities) and a “red envelope lucky draw” (players receive a random amount of money) in the WeChat group) to eliminate any negative reactions caused by participation.

### Results

#### Accuracy in the “Comprehensive Cognitive Ability Test”

The descriptive statistics for the accuracy rate (the percentage of the number of correct answers answered by the participant relative to the total number of questions) for each section of the “Comprehensive Cognitive Ability Test” in the two groups in the experimental task are shown in Table 1.

**TABLE 1:** Accuracy in the “Comprehensive Cognitive Ability Test” (Mean and *SD*).

	Frustration group (*n* = 48)	Fulfillment group (*n* = 22)
Math	0.277 ± 0.137	0.364 ± 0.126
Logic	0.388 ± 0.132	0.618 ± 0.144
Literature	0.475 ± 0.136	0.677 ± 0.151

An independent samples *t* test was performed on the accuracy of the answers for each section of the test (mathematics, logic, and language comprehension) between the two groups. Participants in the frustration group had significantly lower scores on each part [*t*_*Mathematics(68*__)_ = −2.514, *p*_*Mathematics*_ = 0.014, Cohen’ s *d* = 0.661, 1-*β* = 0.716; *t*_*Logic(68*_*_)_* = −6.621, *p*_*Logic*_<0.001, Cohen’ s *d* = 1.665, 1-*β* = 0.999; *t*_*Comprehension(68*__)_ = −5.579, *p*_*Comprehension*_<0.001, Cohen’ s *d* = 1.406, 1-*β* = 0.999]. This shows that there were significant differences in the performance of the two groups on their respective tasks. The task performance of the frustration group (high difficulty task with negative feedback) was significantly lower compared with that of the fulfillment group (low difficulty task with positive feedback).

#### Chinese Mood Adjective Check List

The descriptive statistics of CMACL in each dimension are shown in Table 2.

**TABLE 2:** Chinese mood adjective check list scores (Mean and *SD*).

	Frustration group (*n* = 48)	Fulfillment group (*n* = 22)
Fidgeting (F)	28.27 ± 6.86	19.77 ± 7.52
Happy and excited (HE)	10.88 ± 4.86	20.59 ± 5.19
Pained and sad (PS)	17.92 ± 5.23	14.73 ± 5.51
Angry and hating (AH)	18.92 ± 5.22	16.41 ± 5.82

An independent samples *t* test was performed on the scores of each dimension of the CMACL to explore the differences between the two groups. There were significant differences between the two groups for the F, HE, and PS dimensions. The frustration group reported higher levels of fidgeting [*t*_(68_*_)_* = 4.67, *p* < 0.001, Cohen’ s *d* = 1.181, 1-*β* = 0.995]; pain and sadness [*t*_(68)_ = 2.33, *p* = 0.023, Cohen’ s *d* = 0.594, 1-*β* = 0.623]; anger and hatred [*t*_(68)_ = 1.80, *p* = 0.076, Cohen’ s *d* = 0.454, 1-*β* = 0.412]; and lower levels of happiness and excitement [*t*_(68__)_ = −7.60, *p* < 0.001, Cohen’ s *d* = 1.931, 1-*β* = 0.999]. Thus, participants in the frustration group were more likely to experience negative feelings such as fidgeting and sorrow and less likely to experience positive feelings such as pleasure and excitement than those in the fulfillment group. In other words, the induced frustration manipulation was found to significantly induce negative emotions and suppress positive emotions.

#### Self-Reported Frustration and Fulfillment

After completing the experimental task, participants self-assessed the frustration and fulfillment they experienced after the experiment. The descriptive statistics are shown in Table 3.

**TABLE 3:** Self-reported frustration and fulfillment (Mean and SD).

	Frustration group (*n* = 48)	Fulfillment group (*n* = 22)
Frustration	3.63 ± 0.84	2.32 ± 1.17
Fulfillment	1.67 ± 0.72	3.50 ± 0.80

An independent samples *t* test was performed on the self-reported scores for frustration and fulfillment to explore differences between the two groups. There were significant differences between the two groups for both frustration and fulfillment. The frustration group rated higher scores for frustration [*t*_(68)_ = 4.71, *p* < 0.001, Cohen’ s *d* = 1.286, 1-*β* = 0.998] and lower scores for fulfillment [*t*_(68__)_ = −9.51, *p* < 0.001, Cohen’ s *d* = 2.405, 1-*β* = 0.999]. This confirmed that the experimental manipulation consisting of a high difficulty task with negative feedback could significantly induce frustration.

## Experiment 2: The Effect of Frustration on Altruistic Behavior Tendency and Level in College Students

Experiment 1 verified that negative feedback and a high difficulty task (as completed by the frustration group) can induce frustration. Experiment 2 used the same frustration-inducing task and the same experimental design (fulfillment vs. frustration group) and assessed fund-raising donations from participants’ reimbursements after the experiment to explore whether there was a difference in the tendency and level of altruistic behavior between the two groups. Specifically, the effects of frustration on altruistic tendency and level were tested in Experiment 2, where both Hypothesis 1b and Hypothesis 2b were tested.

### Method

The ethics committee at the sponsoring university approved all procedures used in the current study. Informed consent was obtained from all participants after thoroughly explaining the nature and consequences of this study. Each participant consented to the submission of the findings for publication.

#### Participants

A total of 60 undergraduates (35 in the frustration group and 25 in the fulfillment group) at Nanjing University of Chinese Medicine were enrolled as participants. Participants were randomly assigned to groups. Data from participants who repeated the same choice more than five times or quitted the experiment halfway were deleted. Fifty-four participants had valid data (29 vs. 25), which resulted in 15 males in the frustration group and 9 males in the fulfillment group. The participants aged 17–21 years old (*M* = 18.89 years, *SD* = 0.88 years).

#### Procedure

Experiment 2 adopted a single factor between-subjects design. The independent variable was the combination of artificial feedback and task difficulty, the same as in Experiment 1. There were two levels: high difficulty task with negative feedback (frustration group), and low difficulty task with positive feedback (fulfillment group). The two dependent variables were whether the participants donated (tendency toward altruistic behavior) and the amount of donation (level of altruistic behavior).

Experiment 2 was programmed and conducted using E-prime 2.0.8, and statistical analysis of the data was performed using SPSS 22.0 software. Experiment 2 consisted of three parts as described below.

#### Frustration-Inducing Experimental Task

The two groups were required to complete the predesigned “Comprehensive Cognitive Ability Test,” using the same materials and experimental methods as in Experiment 1.

#### Altruistic Behavior Observation

After participants completed the “Comprehensive Cognitive Ability Test,” they were informed that the experiment was over, and 30 yuan was sent to the participants as experimental reimbursement via Alipay transfer. Alipay, which serves more than one billion users and more than 80 million merchants, is the largest mobile payment platform in China^1^. All participants in this study use Alipay on a daily basis. After the participants confirmed receipt, they signed the remittance confirmation and left the laboratory. Another experimenter was placed outside the door and distributed the fabricated “charity” leaflet to the participants, suggesting that they take it away and read it. The leaflet informed the participants that this experimental project had a cooperative relationship with a charitable organization and asked if they were willing to anonymously donate part of their reimbursement to support public welfare undertakings. They were given a QR code for the Alipay collection disguised as a charitable organization. The participants had 2 h to choose whether to donate to the “charity” on the leaflet and if so, how much to donate. By referring to the Alipay bill and the account information of the transferee, the researcher recorded whether each participant donated and if so, the amount of the donation.

#### Debrief

Two hours after the participants completed the experiment, the researchers debriefed them on the true purpose of the experiment to comply with the relevant ethics (according to 1964 Declaration of Helsinki and its later amendments or comparable ethical standards) through WeChat, and stopped recording any subsequent donation data. For the participants who quit the experiment voluntarily halfway, we immediately conducted debriefing.

The same debriefing procedure as used in Experiment 1 was used. In addition, we informed the participants that the real purpose of the experiment was to compare the donation behavior of the participants under the different circumstances, and all of the money they donated would be donated to the Red Cross Society of China Jiangsu Branch (a well-known local charity).

### Results

#### Tendency Toward Altruistic Behavior

The number of participants in each group and whether they donated is shown in Table 4.

**TABLE 4:** Number of participants with tendency toward altruistic behavior by group.

	With donation behavior (*n* = 13)	Without donation behavior (*n* = 41)
Frustration group	7	22
Fulfillment group	6	19

A chi-squared test was conducted on the number of participants in the two groups regarding donating behavior. There was no significant difference in the number of participants who chose to make donations after the experiment between the frustration group and the fulfillment group (*χ*^2^_(1)_< 0.001, *p* = 0.991). This suggests that whether participants donated was independent of whether they had experienced frustration.

#### Level of Altruistic Behavior

The descriptive statistics for the percentage of reimbursement that the participants donated by experimental group are shown in Table 5.

**TABLE 5:** Level of altruistic behavior (Mean and *SD*) by group.

Frustration group (*n* = 7)	Fulfillment group (*n* = 6)
80.0% ± 34.2%	36.2% ± 16.2%

An independent samples *t* test was performed on the percentage of reimbursement (donation amount per person) by the participants who donated between the two groups. The donation amount by the participants in the frustration group (as a percentage of their reimbursement) was significantly higher than that of the fulfillment group [*t*_(8.834)_ = 3.013, *p* = 0.015, Cohen’ s *d* = 1.637, 1-*β* = 0.999]. This suggests that when making donations, a frustrating situation induced donors to make larger donations.

## Replicability of the Experiments

Replicability is incredibly important for scientific research. In the current study, a repeated experiment (*n* = 37) was performed for both Experiment 1 and Experiment 2. A total of 40 undergraduate students (17–21 years old, *M* = 18.78 years, *SD* = 0.89 years) from Nanjing University of Chinese Medicine (20 in the frustration group and 20 in the fulfillment group) were enrolled. There were 3 participants (2 females) in the frustrated group that did not complete the experiment (dropping out halfway). As a result, there were 17 participants (8 females) in the frustration group and 20 participants (10 females) in the fulfillment group. All the procedures and data processing methods in the repeated experiment were the same as those for the original Experiment 1 and Experiment 2.

In the repeated experiment, the results of the independent *t* test on the scores of each dimension of the CMACL, self-reported frustration, and self-reported fulfillment were consistent with the results of Experiment 1 (Table 6).

**TABLE 6:** Results of *T*-tests in the repeated experiment and experiment 1.

Condition	Experiment	*t*	*df*	*P*
Fidgeting (F)	Experiment 1 (*n* = 70)	4.67	68	<0.001
	Repeated experiment (*n* = 37)	2.52	35	0.017
Happy and excited (HE)	Experiment 1 (*n* = 70)	−7.60	68	<0.001
	Repeated experiment (*n* = 37)	−3.39	35	0.002
Pained and sad (PS)	Experiment 1 (*n* = 70)	2.33	68	0.023
	Repeated experiment (*n* = 37)	2.44	35	0.020
Angry and hating (AH)	Experiment 1 (*n* = 70)	1.80	68	0.076
	Repeated experiment (*n* = 37)	1.66	35	0.106
Frustration	Experiment 1 (*n* = 70)	4.71	68	<0.001
	Repeated experiment (*n* = 37)	3.08	35	0.04
Fulfillment	Experiment 1 (*n* = 70)	−9.51	68	<0.001
	Repeated experiment (*n* = 37)	−4.39	35	< 0.001

Regarding donation behavior, the amount donated by the participants in the frustration group (as a percentage of their reimbursement) was significantly higher than that for the fulfillment group (see Table 7).

**TABLE 7:** Level of altruistic behavior (Mean and *SD*) by group.

	Frustration group	Fulfillment group
Experiment 2	80.0% ± 34.2%	36.2% ± 16.2%
Repeated experiment	70.7% ± 29.3%	36.7% ± 13.9%

An independent sample *t* test was performed on the percentage of the reimbursement donated (i.e., donation amount per person) by the participants who donated between the two groups, and the results of the repeated experiment [*t*_(8)_ = 2.344, *p* = 0.047] were congruent with those of the original iteration of Experiment 2 [*t*_(8.834)_ = 3.013, *p* = 0.015].

Due to the limitations imposed by the COVID-19 pandemic, this study only recruited a small number of participants when conducting the repeated experiment; the research results might also be more meaningful if additional variables (e.g., participants’ demographic characteristics, culture, and type of altruistic behavior) were considered. Regardless of these limitations, it is still worth noting that the results of the repeated experiment were consistent with the original results for Experiment 1 and Experiment 2, which supports, to a certain extent, the reproducibility of the study methods.

## Discussion

### Effectiveness of Frustration-Inducing Experiment

In Experiment 1, participants in the two groups achieved different results in their respective “Comprehensive Cognitive Ability Test” tasks, with the performance of the frustration group being significantly poorer than the fulfillment group, supporting Hypothesis 1a. Participants also differed in their subsequent emotional states according to group. The frustration group reported higher frustration, higher negative emotional experiences such as fidgeting, pain and sorrow, and reduced positive emotional experiences such as pleasure and excitement, compared to the fulfillment group. Thus, Hypothesis 2a was partly supported, and Hypothesis 3a was fully supported.

Experiment 1 demonstrated that this paradigm could effectively induce frustration. However, in both Experiment 1 and Experiment 2, some participants in the frustration group (eleven participants in Experiment 1 and four participants in Experiment 2) left the laboratory before completing the task, a decision that may have been driven by strong frustration induced by the experiment. Individuals have different levels of mental resilience and different attitudes towards experimental tasks, so despite receiving the same experimental treatment, the degree of frustration induced in participants may be different. The issues of how to effectively manipulate the degree of frustration experienced by participants such that it is similar, and how to reduce early withdrawal from such experiments are still to be studied.

### Novelty of Frustration-Inducing Experiment

The results from Experiment 1 showed that the experimental task with high difficulty questions and negative feedback is capable of inducing frustration. In other words, it is possible to provoke immediate frustration in participants experimentally by creating difficulties while they are performing a test assessing different aspects of their ability and leading them to believe they are performing poorly in comparison to others.

The novelty of this method is that, unlike an artificial manipulation, the feedback regarding accuracy for each question was genuine, which feasibly made the participants in the frustration group feel a sense of incompetence during the experiment. In terms of the pre-set negative feedback, instead of directly pointing out the poor performance of the participants, the frustration-inducing task in the current research used multiple presentations of objective negative information (e.g., your mathematical ability exceeds 14.7% of peers) and concluded with the poor performance of the participant at the end (Your Comprehensive Cognitive Ability: Poor) to induce frustration among participants. This kind of manipulation can increase the authenticity of the experiment, and let the subjects feel that the results of the task were explicable. The participants were also asked to evaluate their own ability during each part before the experiment to induce frustration through the contrast between their ideal and the achieved feedback.

Previous studies often used tasks that have little to do with the subjects’ own needs, such as solving puzzles or counting figures, as well as used only a small number of rewards after successful completion as motivation for the subjects to complete the tasks (Lin, 2017). The motivational effects of such rewards are questionable, and previous studies have rarely verified the effects of frustration-inducing tasks. Therefore, this study provides a new method for frustration-inducing tasks—that is, using tasks related to the participants’ own abilities (which may be more relevant to the participants) and, through high difficulty and negative feedback, was able to induce frustration effectively.

### The Influence of a Frustrating Situation on Altruistic Tendency and Level

In Experiment 2, there was no statistically significant difference in the altruistic tendency (donation behavior) between the two groups, and the number of participants who made donations in the two groups did not differ statistically. However, among the participants who had donated, there was a significant difference in the altruistic level (amount of donation) between the two groups, with those in the frustration group donating more. This result is consistent with Hypothesis 2b that frustration can induce a higher level of altruistic behavior. However, in relation to the tendency toward altruistic behavior, findings indicated that college students’ altruistic behavioral tendencies are not affected by whether they were in a frustrating situation. In other words, the experimental results are not able to confirm Hypothesis 1b.

This finding may be due to the fact that the impact of frustration on individuals in the current experiment was relatively short and superficial, and hence, it was difficult to promote a change in individuals’ altruistic personalities, and this is reflected by the results in that there was no significant difference in the number of participants who donated between the two groups. However, among the participants who made donations, the amounts donated between the two groups differed significantly, which in turn implied that frustration had an impact on the level of altruistic behavior. In addition, the results of Experiment 1 showed that individuals who experience frustration are more likely to experience anger and sorrow at the same time, and experience less happy and excited emotions. From this perspective, the emotions induced by frustration and trauma are similar (i.e., both are negative) but the severity is likely to differ. For example, sadness expressed by an undergraduate on leaving a challenging experiment is likely to differ in intensity and duration to that of an individual having lost family members in a horrific accident.

Besides frustration, many factors may affect altruistic behavior. For example, the amount of money, which was not explored in this study, may affect altruistic behavior. In a meta-analysis (*n* > 12000) conducted by Fromell et al. (2020) regarding the Dictator Game as an effective method to measure altruism (Capraro, 2019), found no significant association (*p* = 0.522) between stake level (values participants received in the decision situation) and the effect of intuition on altruism (Fromell et al., 2020). The current research was based on a real situation, and the relationship between the values obtained by the participants and the altruistic behavior still needs further study.

### Limitations of the Research

The factors that influence the decision of whether to engage in altruistic behavior are plentiful and complicated. Although the experimental results show that a frustrating situation can significantly increase the amount of donation that donors make (that is, the level/degree of altruistic behavior), there is no significant difference in the tendency toward altruistic behavior between the two groups of participants in Experiment 2. It is important to note that in Experiment 2, participants were not in the laboratory when making their anonymous charitable donation decisions. This increased the external validity but simultaneously decreased the internal validity of the experiment as there is no guarantee that all participants read the donation instructions or made donation decisions in the same environment. When participants read the “charity leaflets” distributed by the experimenters, it was difficult to know whether they were with peers or other participants. Therefore, there may be a peer effect on the donation decision in that the level of donation of other donors will also affect the individual’s donation decisions (Griskevicius et al., 2008). Experiment 2 of this study was a between-subjects design, so it may be considered that the peer effects of the two groups would be equivalent. However, future research should further explore the relationship and potential interaction between peer effects and frustration.

In this study, only altruistic behaviors such as “charitable donation behaviors” were considered, so the findings may have limitations in terms of generalizability. “Charitable donations” is a common and typical altruistic behavior, so presents an appropriate method by which to test the relationship between frustration and altruistic behavior. Whether the impact of frustration on other kinds of altruistic behaviors is consistent with donation behavior still requires study.

In the design of the experiment, social desirability effects were controlled for. However, although we ensured external validity, the internal validity decreased since we did not control the environment of every participant when deciding whether to conduct donation behavior. This has both advantages and disadvantages, and future research should pay more attention to the internal validity of the naturalistic observation method (i.e., designing a more rigorous and realistic experimental environment or identifying an alternative way to observe donation behavior).

The problem of the small sample sizes in this research is also a limitation, and only undergraduates were recruited, potentially limiting the generalizability of the results. It would be worthwhile in future research to explore whether occupation, age, income, and many other demographic variables have effects on individuals’ altruistic behavior. Moreover, although this study found the phenomenon that the frustrated participants were inclined to donate more money when making donations, the underlying mechanism of such phenomenon was not explained by the experiments. Instead of simply manipulating a few variables, designing experiments as a progressive or complementary relationship is more judicious.

### Implications of the Research

In addition to exploring the positive impact that frustration may have on individuals, the current study provides an experimental supplement to the research on altruism in dual-process theory. The difficult frustration-inducing task in the current study, which may inhibit the use of deliberation (System 2) and promote the use of intuition (System 1), resulted in a higher level of altruism for some participants; this result is partially consistent with previous studies (Evans and Stanovich, 2013; Capraro, 2019; Fromell et al., 2020). This means that, despite experiencing frustration, some participants were still more likely to choose intuition as strategy when considering altruism. It is worth noting that individual differences exist in the process of selecting strategies (Rand et al., 2016), and this phenomenon did appear in the current study (not everyone chose System 1 or engaged in altruistic behavior). This study partially supports the view that a high cognitive load may lead to greater likelihood of adopting intuition as a strategy, and found that frustration had no significant impact on individual strategy choices. Future research could take emotional state into consideration to better improve our understanding of dual-process theories.

With the rapid development of the world economy, the pressure on college students and their need for self-realization has gradually increased, which in turn has pushed college students to be more likely to encounter setbacks (Li et al., 2017). Thus, a comprehensive understanding of frustration for the prevention of mental health problems and to improve students’ ability to resist setbacks is necessary. According to a survey conducted in a university in China, only 15.6% of students said they had not experienced frustration (Zhang et al., 2010). A previous study indicates that “despite relatively high levels of psychological distress, many students in higher education do not seek help for difficulties (Laidlaw et al., 2016).” Hence, research on frustration among individuals, especially undergraduates, is meaningful, and is why undergraduates were selected as participants in the current research. For a long time, frustration has been considered negative. Nevertheless, as it was found in this study, frustration sometimes can increase the altruistic tendency of participants, which is considered to be a positive impact. In practice, instead of minimizing frustration by all means, practitioners can guide frustrated clients to discover the positive impact of frustration, and to understand frustration from a comprehensive perspective. In this way, the frustrated clients may realize that what they are suffering is not downright negative because good deeds could come from frustrated individuals. Moreover, educators can encourage students to explore the unknown world because both the joy after success and the frustration after failure have positive meanings. For all of us, it is high time to revisit “frustration.” After all, to promote a more peaceful world, it is important to understand how victims become caring rather than aggressive (Staub and Vollhardt, 2008).

As for future research, whether frustration could lead to other forms of short-term personal growth, such as a higher level of empathy, is worthy of study. After all, just like every coin has two sides, negative effects such as aggression are only one side of the result of frustration, and there may be positive effects to be found on the other side.

## Data Availability Statement

The original contributions presented in the study are included in the article/Supplementary Material, further inquiries can be directed to the corresponding author.

## Ethics Statement

The studies involving human participants were reviewed and approved by Ethics Committee of Nanjing University of Chinese Medicine. Written informed consent to participate in this study was provided by the participants’ legal guardian/next of kin.

## Author Contributions

YP wrote the first draft of the manuscript and analyzed the data. YP and JT contributed the conception and design of the study. JT checked and corrected the scientific issue of the study. YP and HL conducted the experiment. All authors contributed to the manuscript revision and approved it for publication.

## Conflict of Interest

The authors declare that the research was conducted in the absence of any commercial or financial relationships that could be construed as a potential conflict of interest.

## Footnotes

1 Data source URL: https://xueqiu.com/7920634912/157915891
